# Involvement in the US criminal justice system and cost implications for persons treated for schizophrenia

**DOI:** 10.1186/1471-244X-10-11

**Published:** 2010-01-28

**Authors:** Haya Ascher-Svanum, Allen W Nyhuis, Douglas E Faries, Daniel E Ball, Bruce J Kinon

**Affiliations:** 1 Eli Lilly and Company, Indianapolis, IN, USA

## Abstract

**Background:**

Individuals with schizophrenia may have a higher risk of encounters with the criminal justice system than the general population, but there are limited data on such encounters and their attendant costs. This study assessed the prevalence of encounters with the criminal justice system, encounter types, and the estimated cost attributable to these encounters in the one-year treatment of persons with schizophrenia.

**Methods:**

This post-hoc analysis used data from a prospective one-year cost-effectiveness study of persons treated with antipsychotics for schizophrenia and related disorders in the United States. Criminal justice system involvement was assessed using the Schizophrenia Patients Outcome Research Team (PORT) client survey and the victimization subscale of the Lehman Quality of Life Interview (QOLI). Direct cost of criminal justice system involvement was estimated using previously reported costs per type of encounter. Patients with and without involvement were compared on baseline characteristics and direct annual health care and criminal justice system-related costs.

**Results:**

Overall, 278 (46%) of 609 participants reported at least 1 criminal justice system encounter. They were more likely to be substance users and less adherent to antipsychotics compared to participants without involvement. The 2 most prevalent types of encounters were being a victim of a crime (67%) and being on parole or probation (26%). The mean annual per-patient cost of involvement was $1,429, translating to 6% of total annual direct health care costs for those with involvement (11% when excluding crime victims).

**Conclusions:**

Criminal justice system involvement appears to be prevalent and costly for persons treated for schizophrenia in the United States. Findings highlight the need to better understand the interface between the mental health and the criminal justice systems and the related costs, in personal, societal, and economic terms.

## Background

Individuals with severe mental illness are over-represented in the criminal justice system when compared with the larger US population. In the United States each year, approximately 1 million detentions in county jails involve persons with serious mental illnesses. These individuals are imprisoned about 8 times more frequently than they are admitted to state mental hospitals [1] and are incarcerated for significantly longer time than other inmates [2].

Although persons with schizophrenia are known to have a higher risk of arrest and incarceration compared with the general population [3], many of their other legal contacts result from being victimized by others rather than from unlawful behavior on their part [4].

Studies of the cost of schizophrenia in the United States have typically not included costs associated with legal encounters [5-9], although data from non-US studies suggest that encounters with the criminal justice system constitute a substantial proportion of indirect costs [10,11]. One study estimated that the overall annual (2002) US cost of schizophrenia includes $2.64 billion in direct non-health care costs for law enforcement [12].

Although prior research has assessed the mental health status of persons already incarcerated within the criminal justice system, little is known about schizophrenia patients' involvement in the US criminal justice system, the type of legal encounters they have, and the related direct economic cost. To that end, a post-hoc analysis was conducted of data from a one-year cost-effectiveness naturalistic study of patients treated with antipsychotics for schizophrenia in the United States. That study assessed several types of encounters the patients had with the criminal justice system, which enabled us to identify the prevalence of such encounters, characterize the specific types of legal encounters, estimate their direct costs, compare the health care costs between patients with and without any criminal justice system involvement, and estimate the proportion of the total health care cost attributable to patients' legal involvement.

## Methods

### Data source

This post-hoc analysis used data from an Eli Lilly-sponsored one-year multicenter, prospective, randomized open-label cost-effectiveness study of typical and atypical antipsychotics in the treatment of schizophrenia [13]. Patients who were 18 years or older with a DSM-IV diagnosis of schizophrenia, schizoaffective, or schizophreniform disorder and a score of 18 or more on the Brief Psychiatric Rating Scale [14] were eligible to participate. No patient was excluded because of involvement with the legal system or having concurrent substance abuse disorder or other psychiatric or medical comorbidities.

This study was conducted at 21 sites in 15 states between May 1998 and September 2002. The protocol and consent procedures were approved by institutional review boards in accordance with the Code of Ethics of the World Medical Association (Declaration of Helsinki), and after a complete description of the study was given to the subjects, signed consent forms were obtained from patients prior to participation in the study. Further details about the parent study design and methods have been published [13]. Study participants who had at least 1 assessment of their legal encounters were included in the current analysis. Legal encounter information from the previous 12 months was gathered at baseline and again at 3 post-baseline assessments at 2, 8, and 12 months (endpoint).

### Measures

Patients' baseline sociodemographic and clinical characteristics were assessed using standard psychiatric measures. Sociodemographic characteristics were based on a structured interview, the level of symptom severity was assessed with the Positive and Negative Syndrome Scale (PANSS) [15], and the level of functioning was assessed using the Medical Outcome-Short Form 36 health survey [16]. Resource utilization (e.g., hospitalizations, use of antipsychotics) was assessed via regular and systematic abstraction of patients' medical records with an abstraction form developed for the study. Information about prescribed antipsychotic medications in the 6 months prior to enrollment was used to assess prior medication adherence level. Adherence was defined using a customary proxy measure - the Medication Possession Ratio (MPR) [17] that reflects the proportion of days with any antipsychotic medication in the 6 months prior to study enrollment. In addition to mean MPR (higher is better), the proportion of patients deemed adherent (MPR ≥80%) was also calculated in accordance with prior research [17].

Information from 2 measures was used to assign each participant to a group with or without criminal justice system involvement: 1) the Schizophrenia Patient Outcome Research Team (PORT) client survey [18] and 2) the victimization subscale of the Lehman Quality of Life Interview (QOLI) [19]. The PORT survey of patients' legal involvement and the QOLI items on being a victim of crime provided information on 11 types of encounters resulting in arrest and 7 other types of encounters not leading to arrest. Encounters resulting in arrest included robbery or burglary, vandalism, parole or probation violation, drug charges, forgery, weapon offense, assault, arson, rape, prostitution, and homicide. Encounters not resulting in arrest included contempt of court, disorderly conduct, driving while intoxicated, major driving violations, parole or probation for at least 1 year, being a victim of a crime, and other miscellaneous encounters. The QOLI victimization subscale included 2 items that assessed whether the participant was a victim of any violent crime (e.g., assault, rape, mugging, or robbery) or a non-violent crime (e.g., burglary, theft, or being cheated). This was assessed at baseline (reflecting encounters in the past 12 months) and again at 2, 8, and 12 months or endpoint, reflecting encounters since the previous assessment. To correct for potentially erroneous repetitive participant reports of encounters with the criminal justice system, each type of reported legal involvement was counted only once for each participant. However, if the patient reported having several encounters of different types, the costs of these different encounters were added.

### Cost estimates

Consistent with cost calculation in the parent cost-effectiveness study [13], direct annual health care costs for each participant were estimated in 2001 US dollars using resource utilization abstracted from patients' medical records together with Medicare data as cost benchmarks for units of specific services. Medication costs were based on 2001 average wholesale prices discounted by 15% to better reflect "real-world" costs [13].

Since the parent study did not collect cost information for patients' criminal justice system encounters, the cost of involvement was estimated using previously reported costs per type of encounter with the legal system in a published US study [20] and adjusted the cost to reflect 2001 US dollars to be consistent with the year in which health care costs were estimated in the parent study. In addition, the annual cost of probation was estimated to be $3,236.51, based on a report by the US Office of Probation and Pretrial Services [21], adjusted to 2001 dollars.

### Statistical analysis

Analyses compared participants who reported no legal involvement with those reporting any involvement at baseline or at any of the 3 post-baseline assessments. Chi-square, Fisher's exact, and *t *tests were used for univariate comparisons of demographic, clinical, and functional baseline variables. In addition to unadjusted group comparisons on these variables, adjusted comparisons were conducted using a logistic regression analysis with adjustment for age, gender, and ethnicity. Comparisons of mean costs between participants with versus without criminal justice system involvement used nonparametric bootstrapping stratified by propensity scoring to adjust for age, gender, and ethnicity. The nonparametric Wilcoxon rank-sum test was used to make univariate comparisons of annual cost variables. All tests were two-tailed at α = 0.05.

## Results

### Patient characteristics

Of the 651 participants, 42 were excluded from this analysis because of incomplete PORT client survey data, leaving 609 patients. Almost all of these participants (581 of 609, or 95%) were outpatients at baseline.

Nearly one-half of the patients (278 or 46%) reported at least 1 legal encounter at baseline or during any post-baseline assessment. At baseline, participants with criminal justice system involvement were significantly younger (p < .001), had a poorer level of mental health functioning (p < .001), were more likely to have been hospitalized in the prior year (p = .003), to use emergency services (p = .021), to have a lifetime diagnosis of substance abuse disorder (p < .001), to drink alcohol to intoxication (p = .049), and to use cannabis and cocaine (p < .001). They were also significantly less likely to be adherent to antipsychotics (MPR ≥80%) (p = .001), to have a lower mean MPR level (p < .001), and to drop out of the one-year study (p = .020) (Table 1). Participants with any criminal justice system encounter did not significantly differ from those without encounters on gender, ethnicity, uninsured status, illness severity per PANSS total score, and on physical level of functioning. Following adjustment for age, gender, and ethnicity, results remained essentially the same on 13 of 17 key patient characteristics. The 4 variables that changed in significance level indicated that the group with criminal justice system involvement had a significantly poorer level of physical functioning (p = .037), while 3 previously (unadjusted) significant group differences on use of emergency services, drinking alcohol to intoxication, and study discontinuation rates became statistically non-significant. All other group comparisons on key characteristics were essentially unchanged.

**Table 1:** Key characteristics of participants with and without legal system involvement

Variable	With legal involvement (n = 278)	% of total sample	Without legal involvement (n = 331)	% of total sample	Unadjusted p value	Adjusted p value^a^
Age, mean ± SD, yr	41.0 ± 11.7		44.5 ± 12.1		< .001	.001
Male	180	65%	200	60%	.277	.776
Ethnicity					.836	.999
Caucasian	149	54%	185	56%		
African-American	95	34%	109	33%		
Other	34	12%	37	11%		
Uninsured	57	21%	48	15%	.051	.238
PANSS total score, mean ± SD	86.4 ± 18.3		86.9 ± 21.2		.770	.772
SF-36 Mental composite score^b ^± SD	-1.3 ± 1.3		-0.9 ± 1.3		< .001	.001
SF-36 Physical composite score^b ^± SD	-0.5 ± 1.1		-0.4 ± 1.0		.218	.037
Not hospitalized in the previous year	172	64%	245	75%	.003	.034
ER use in the previous 3 months	87	32%	76	24%	.021	.056
Lifetime substance abuse diagnosis	145	52%	117	35%	< .001	< .001
Drink alcohol to intoxication	29	11%	19	6%	.049	.088
Cannabis use	60	22%	19	6%	< .001	< .001
Cocaine use	23	8%	6	2%	< .001	.001
MPR ≥ 80%	92	36%	146	50%	.001	.015
MPR, mean ± SD	0.5 ± 0.4		0.6 ± 0.4		< .001	.001
Discontinued study	84	30%	72	22%	.020	.053
Mean days to study discontinuation, mean ± SD	312.6 ± 93.9		325.3 ± 84.2		.082	.136

Abbreviations: ER, emergency room; MPR, medication possession ratio; PANSS, positive and negative syndrome scale; SD, standard deviation; SF-36, Medical Outcome--Short Form 36. Possible PANSS scores range from 30 to 210, with higher scores indicating greater pathology. SF-36 standardized scores are relative to the general population, where smaller negative scores indicate better health.

^a^Adjusted p value for age, gender, and ethnicity.

^b^Weighted averages of the Z-scores of the 8 Medical Outcome--Short Form 36 domain scores as related to general population norms.

### Types of legal encounters

Among the 18 types of criminal justice system encounters assessed in this study, being a victim of crime was the most prevalent type of involvement (67%), followed by being on parole or probation (26%), arrest for assault (13%), other miscellaneous encounters not resulting in arrest (13%), being cited for a major driving violation (e.g., reckless driving) but without arrest (11%), arrest for parole or probation violation (10%), and being charged with disorderly conduct (9%). All other types of encounters had a prevalence rate lower than 5% (Table 2).

**Table 2:** Types of encounters with criminal justice system, their prevalence rates, and estimated costs

Category	Persons with the criminal justice system encounter (n = 278)	% of total sample	Mean ± SD cost per encounter, $^a^
**Crime Against the Subject**			

Victim of crime	187	67%	60 ± 79

**Encounters resulting in arrest**			
On parole or probation for 1 year	73	26%	3237 ± NA
Assault	35	13%	1572 ± 1358
Parole or probation violation	27	10%	2751 ± 4820
Drug charges	11	4%	2077 ± 3817
Burglary, larceny, breaking and entering	11	4%	2783 ± 4914
Shoplifting, vandalism	8	3%	2783 ± 4914
Weapons offense	7	3%	1599 ± NA
Robbery	5	2%	2783 ± 4914
Arson	6	2%	2751 ± 4820
Rape	4	1%	19278 ± 22463
Prostitution	2	1%	2332 ± 2518
Homicide or manslaughter	4	1%	19278 ± 22463
Forgery	2	1%	447 ± 237

**Encounter not resulting in or from arrest**			
Other miscellaneous encounter	36	13%	38 ± 87
Major driving violation	30	11%	1016 ± 179
Disorderly conduct	25	9%	1246 ± 896
Driving while intoxicated	6	2%	1016 ± 179
Contempt of court	6	2%	38 ± 87

^a^Estimated using previously reported costs per type of encounter with the legal system [20] and adjusted to 2001 US dollars.

Abbreviation: NA, not available; SD, standard deviation.

^a^Cost per encounter was adopted from Clark and colleagues [20] and adjusted to 2001 dollars. Cost of parole or probations for 1 year was adopted from the US Office of Probation and Pretrial Services and adjusted to 2001 dollars.

### Cost analyses

Overall, the mean estimated annual per-patient cost of criminal justice system involvement was $1,429, using 2001 dollars. Compared to participants free of encounters, the annual direct health care costs for participants with any encounter were numerically higher (but not statistically significantly different - unadjusted and adjusted) for total direct annual health care costs ($23,121 vs. $20,206; p = .346), with no significant cost differences on outpatient services, medication, or labs and other costs. The cost of inpatient hospitalization was, however, significantly higher for participants with any encounter ($10,330 vs. $5,376; p = .001 unadjusted, p = .019 adjusted) (Table 3). The mean annual per-patient cost of criminal justice system involvement ($1,429, SD = $2,676) translated to 6% of the annual total direct health care costs for participants with involvement (Table 3). Because being a victim of crime was the most prevalent type of encounter and among the least costly encounters, the proportion of the total cost attributable to these encounters was also calculated after excluding participants whose only legal encounter was being a victim of crime (113 or 41% of participants with any encounter). As a result, the mean annual per-participant cost of criminal justice system involvement increased from $1,429 to $2,386 (SD = $3,135), comprising 11% (instead of 6%) of the total health care cost per participant ($22,002, SD = $33,660).

**Table 3:** Annual direct health care cost and cost components for persons with versus without any criminal justice system encounters

	Mean ± SD, $			
			
Cost category	Persons with any criminal justice system involvement (n = 278)	Persons without any criminal justice system involvement (n = 331)	Unadjusted p value	Adjusted p value^a^
Total health care cost	23121 ± 31813	20206 ± 28307	.346	.420
Inpatient hospitalization	10330 ± 26226	5376 ± 18525	.001	.019
Outpatient care	6890 ± 16594	8503 ± 18727	.424	.150
Medication	4704 ± 3045	5001 ± 3531	.508	.183
Labs and other cost	1197 ± 3990	1326 ± 3272	.286	.672

Abbreviation: SD, standard deviation.

^a^Adjusted p-value for age, gender, and ethnicity.

### Victims of crime versus perpetrators

Considering that being a victim of crime was found to be the most prevalent type of legal involvement in the study (67%), it was of interest to assess differences on key characteristics between victims of crime, perpetrators (who were not victims of crime), and patients without legal involvement. Patients who were both perpetrators and victims of crime (n = 74) were excluded from this analysis to help contrast the 3 groups. Results are presented in Table 4, showing that differences among the 3 groups were very similar to those found between patients with versus without legal involvement, as victims of crime and perpetrators did not significantly differ on any key characteristic except one: perpetrators were significantly more likely to be male (p = .023 unadjusted; p = .046 adjusted).

**Table 4:** Key characteristics of participants without legal system involvement, participants who were only victims of crime, and perpetrators who were not victims of crime.

Variable	A Patients without any legal involvement N = 331	B Patients who were only victims of crime N = 113	C Perpetrator who were not victims of crime N = 91	P-value Unadjusted, overall group comparison (A vs B vs C)	P-value Adjusted for covariates, overall group comparison (A vs B vs C)	P-value Adjusted for covariates, Victims VS Perpetrators (B vs C)
Age, mean (SD)	44.5 (12.1)	44.3 (11.1)	40.0 (12.0)	0.005	0.024	0.066

Male gender, n (%)	200 (60%)	57 (50%)	63 (69%)	0.023	0.046	0.016

Ethnicity -- Caucasian, n (%)	185 (56%)	69 (61%)	45 (49%)	0.566	0.646	0.333

-African-American, n (%)	109 (33%)	33 (29%)	36 (36%)			

-Other ethnic group, n (%)	37 (11%)	11 (10%)	13 (14%)			

Uninsured, n (%)	48 (15%)	13 (12%)	25 (28%)	0.006	0.132	0.074

PANSS total, mean (SD)	86.9 (21.2)	86.9 (18.2)	84.8 (19.0)	0.659	0.648	0.482

SF36 MCS, mean (SD)	-0.85 (1.31)	-1.23 (1.33)	-1.10 (1.32)	0.018	0.025	0.275

SF36 PCS, mean (SD)	-0.40 (1.02)	-0.55 (1.14)	-0.39 (1.00)	0.372	0.366	0.990

Past year psych hospitalization None, n (%)	245 (75%)	79 (73%)	53 (60%)	0.016	0.167	0.714

ER past 3 months, n (%)	76 (24%)	32 (29%)	30 (33%)	0.146	0.257	0.454

Lifetime diagnosis of substance use disorder, n (%)	117 (35%)	51 (45%)	45 (49%)	0.024	0.032	0.837

Use of alcohol to intoxication, n (%)	19 (6%)	7 (6%)	9 (10%)	0.362	0.547	0.547

Any use of cannabis, n (%)	19 (6%)	15 (13%)	20 (22%)	< 0.001	< 0.001	0.466

Any use of cocaine, n (%)	6 (2%)	4 (4%)	8 (9%)	0.005	0.018	0.242

MPR ≥ 80%, n (%)	146 (50%)	47 (46%)	28 (34%)	0.042	0.195	0.513

MPR, mean (SD)	0.60 (0.42)	0.53 (0.44)	0.42 (0.44)	0.004	0.023	0.520

Discontinued study, n (%)	72 (22%)	30 (27%)	28 (31%)	0.170	0.294	0.820

Mean days to study discontinuation, mean (SD)	325.3 (84.2)	310.1 (96.0)	312.1 (99.7)	0.200	0.207	0.587

### Patients with versus without legal involvement data

A small proportion of the study participants (42 of 651 or 6.5%) lacked data on the PORT, which assessed patients' legal involvement. We compared patients with and without legal involvement data on key baseline characteristics to ascertain how they might have differed. Results are presented in Table 5, showing the 2 groups significantly differed on several key characteristics, with the most glaring one being that almost all (90%) patients without PORT data have discontinued early from the study (p < .001) with a significantly lower treatment duration ( < .001). They were also significantly more likely to be uninsured (p = .017), had poorer levels of mental health functioning (p = .002), higher use of emergency services (p = .043), more lifetime diagnosis of substance use disorder (p = .024), and poorer levels of adherence to medication regimen (p = .043).

**Table 5:** Key characteristics of participants with and without data on legal system involvement

Variable	A Patients with legal involvement data N = 609	B Patients without legal involvement data N = 42	P-value Unadjusted, overall group comparison (A vs B)
Age, mean (SD)	42.9 (12.0)	41.6 (13.2)	0.480

Male gender, n (%)	380 (62%)	31 (74%)	0.185

Ethnicity -- Caucasian, n (%)	334 (55%)	20 (48%)	0.533

-African-American, n (%)	204 (34%)	15 (36%)	

-Other ethnic group, n (%)	71 (12%)	7 (17%)	

Uninsured, n (%)	105 (18%)	13 (35%)	0.017

PANSS total, mean (SD)	86.7 (19.9)	88.2 (21.2)	0.652

SF36 MCS, mean (SD)	-1.04 (1.32)	-1.71 (1.34)	0.002

SF36 PCS, mean (SD)	-0.44 (1.04)	-0.22 (1.02)	0.189

Past year psych hospitalization: None, n (%)	417 (70%)	24 (60%)	0.216


ER past 3 months, n (%)	163 (28%)	17 (44%)	0.043

Lifetime substance use disorder diagnosis, n (%)	262 (43%)	26 (62%)	0.024

Use of alcohol to intoxication, n (%)	48 (8%)	6 (17%)	0.109

Any use of cannabis, n (%)	79 (13%)	9 (25%)	0.077

Any use of cocaine, n (%)	29 (5%)	2 (6%)	0.694

MPR ≥ 80%, n (%)	238 (44%)	11 (28%)	0.067

MPR, mean (SD)	0.53 (0.43)	0.39 (0.41)	0.043

Discontinued study, n (%)	156 (26%)	38 (90%)	< 0.001

Mean days to study discontinuation, mean (SD)	319.5 (88.9)	123.5 (125.6)	< 0.001

## Discussion

In this post-hoc analysis, about one-half (46%) of the participants reported at least 1 encounter with the criminal justice system, with the 2 most prevalent types of encounters being a victim of any crime (67%) and being on parole or probation (26%). Although this study did not assess the personal or societal burden associated with such encounters, it provides - for the first time - an estimate of the direct economic impact of having encounters with the criminal justice system for persons treated in the United States for schizophrenia over a one-year period, in the context of their total direct health care cost. This study estimated that these encounters may comprise approximately 6% to 11% of the annual per-patient direct total costs. While these estimated costs are applicable to persons already involved with the mental health system, the cost of encounters for persons who are not involved with the mental health system may be substantially higher due to multiple arrests [20] and potentially prolonged incarcerations. Current findings highlight the preponderance of the interface that patients with schizophrenia have with the criminal justice system and its economic cost, 2 important aspects that often go unreported in studies of schizophrenia, although the link between medication nonadherence, violent behaviors, arrests, and being a victim of crime has been previously reported [22-25]. Our findings are consistent with a prior US study, in which 45% of new mental health outpatients had 1 or more criminal justice system encounter before arriving for treatment and offenders were more likely to have drug dependence, to have greater psychological disability, and to have less personal empowerment than other clients [26].

Also consistent with prior research [20,23-27] is our finding that participants with any criminal justice system encounter differed from those without encounters on a number of characteristics. They were younger, with a poorer level of mental health, greater likelihood of substance abuse (alcohol, cannabis, and cocaine), and poorer medication adherence. They were also more likely to have inpatient hospitalizations in the year prior to enrollment, to use emergency services, and to drop out of the study. Although the cause-effect relationship between medication adherence and criminal justice system encounters cannot be delineated from the current study, the findings suggest that involvement may represent yet another, potentially underappreciated, consequence of medication nonadherence [20,23-27].

Current findings also provide new information about the specific types of criminal justice system involvement that patients with schizophrenia tend to have while being treated in usual mental health settings across the United States. Most prior schizophrenia studies have not assessed this type of data. Previous studies have examined prison populations for mental health problems. Our analysis instead evaluated patients with mental health problems who are treated in the community and assessed them for involvement with the criminal justice system. To our knowledge, there is limited data regarding the cost of criminal justice involvement for patients with schizophrenia who are engaged in mental health systems. In fact, we are aware of only one 1999 publication [20] that reported the types and costs of these encounters. That study, by Clark and colleagues, included 203 persons treated for schizophrenia, schizoaffective, and substance use disorders in New Hampshire and used multiple data sources to estimate the direct cost per encounter type and the total cost of patients' legal encounters. They concluded that poor treatment engagement was associated with multiple arrests and consequently greater legal costs, thus highlighting the importance of engaging these often reluctant individuals in effective mental health treatment. To our knowledge, the present study is the first to examine patients with schizophrenia treated in usual care settings and compare those with versus without criminal justice involvement on total health care cost and cost components (cost of inpatient hospitalization), the cost of legal encounters, and the estimated proportion of the total health care cost attributable to patients' legal involvement. Previous studies have examined characteristics of mentally ill patients with versus without involvements with the legal system [26] or examined psychopathology in prison populations, but such studies are of a very different nature and orientation. For example, a recent study of this country's largest state prison system found that individuals with psychiatric disorders were at increased risk of multiple incarcerations [28]. Furthermore, because this post-hoc analysis used data of a cost-effectiveness study in the treatment of schizophrenia, it enabled comparison between participants with versus without criminal justice system encounters on total health care costs and cost components. While there were no statistically significant group differences on total cost and cost of outpatient services, medications, or labs and other costs, the groups significantly differed on inpatient hospitalization cost. The group with any criminal justice system encounter had about twice the inpatient hospitalization cost of those without any encounter, pointing to differential use of the most costly health care resources despite lack of baseline differences in symptom severity level. These findings may reflect the tendency of this group to be more volatile because of substance use behaviors and medication nonadherence. It is worth noting, however, that the higher hospitalization cost in the criminal justice system encounter cohort was consistent with the higher rate of hospitalization in the prior year compared to the group without encounters (36% vs. 25%).

The current findings also underscore the fact that being a crime victim is a highly preponderant phenomenon in this patient population, as it was reported by about two-thirds of the study participants. The link between schizophrenia and victimization has been previously reported and was described as 1 of 6 important adverse outcomes that include violence, suicide/self-harm, substance use, homelessness, and unemployment [29]. The high rate of victimization also highlights how susceptible these patients are to being victims of crime because of their mental disorder and the social context in which they live [30], as they are prone to become prey for criminals because of their disabilities, lacking work, or protected environments, often living in poor, dangerous neighborhoods with high crime rates [31]. These findings suggest that patients with schizophrenia who disclose being crime victims are likely to be at high risk for other adverse outcomes and will require specialized interventions that help address their needs, improve their living arrangements, and increase their engagement in the mental health system. Programs that target these treatment needs (e.g., substance abuse, medication nonadherence) in mentally ill offenders and emphasize treatment services over incarceration of certain individuals have proven beneficial in terms of reducing recidivism and overall criminal justice system expenditures, thus potentially offsetting initial investments in these interventions [1,28,29].

The study has a number of limitations that need to be recognized. This was a post-hoc analysis that will require replication. The study did not collect direct cost data of criminal justice system encounters and estimated the costs using previously reported data. Costs of each type of encounter with the criminal justice system were derived exclusively from a study of dually diagnosed patients (with substance abuse and schizophrenia, schizoaffective, or bipolar disorders) conducted by Clark and colleagues at New Hampshire-Dartmouth Psychiatric Research Center [20] and adjusted to 2001 dollars. This was done because our study did not assess cost of patients' legal encounters, nor are there available data for costing each type of legal encounter across the 15 states in which our study was conducted. This is a clear limitation of the study that hampers the generalization of the findings and thereby helps highlight the need to replicate the findings and to systematically assess direct costs of legal encounters in future research. Despite this limitation and the attendant need for a certain degree of caution in interpreting our findings, we are unaware of any other US study on costs of criminal justice system encounters to serve as a foundation for the economic analyses. Another limitation is our likely underestimation of the true costs associated with patients' legal involvements, because encounters were based on patient self-report (often minimized) and self-report for only 19 types of encounters. In addition, it is possible that some patients were arrested more than once for any one particular offense; however, by our protocol, these incidents were counted only once to avoid potential erroneous repetition. This methodology, together with the fact that we captured patient self-reported victimization and its costs, but not those of arresting or otherwise processing the assailant, would tend to result in an underestimation of the total costs of involvement in the criminal justice system. Another study limitation is the paucity of information about which encounters involved the police and which did not, considering that some encounters (especially victimizations) are not reported to the police and thus do not incur measurable costs. Furthermore, our study consolidated baseline and follow-up involvement with the criminal justice system into 1 measure. However, when we repeated our analyses using only the post-baseline data, the findings were highly consistent with what has been reported in this manuscript (data not shown).

Although the range of assessed encounters was broad, it was not all-inclusive. Missing are costs associated with federal offenses, legal fees, and other forms of involvement with the civil court system (e.g., court commitments, guardianships); domestic violence (which may be a criminal or civil offense); criminal trespass, as well as costs associated with arresting assailants in cases of victimization. Other potential costs, including overtime pay for deputies on suicide watch and daily incarceration costs [2] were not captured. Our analysis may have also over-corrected for patients' propensity toward repetitive reporting of the same encounter over time, because we counted each encounter only once. Although some of the participants may have had repeated encounters during the one-year study, our analysis did not capture them, thus potentially further underestimating the true prevalence and resultant cost attributable to involvement with the criminal justice system. And lastly, the current study included only persons treated for schizophrenia and related disorders at 21 treatment sites across 15 states in the United States and did not include persons diagnosed with schizophrenia who were in jails or prisons. The current findings are considered, therefore, generalizable to most but not all persons diagnosed with schizophrenia.

## Conclusions

Encounters with the criminal justice system are frequent, costly, and perhaps underappreciated outcomes in the treatment of persons with schizophrenia in the United States, where being a victim of crime appears to be the most frequent type of legal involvement. When assessing the costs of schizophrenia, studies should account for potential criminal justice system involvement whenever possible; conversely, analyses that do not take such expenditures into account may underestimate total costs incurred by these patients in the mental health and criminal justice systems. Future well-designed studies that link patient-level resource utilization data from mental health and criminal justice system databases are needed to improve our understanding of the interface between the mental health and criminal justice systems, including the clinical, societal, and economic costs of criminal justice system encounters, among patients with schizophrenia.

## Competing interests

The authors are employees of and minor shareholders (stocks/options) in the study sponsor, Eli Lilly and Company (Indianapolis, IN).

## Authors' contributions

All authors contributed to the study design. DEB and DEF acquired data. All authors interpreted data. HA-S prepared the manuscript with editorial assistance from Stephen W. Gutkin (SWG), Rete Biomedical Communications Corp. (Wyckoff, NJ, USA) with revisions by all authors. All authors read and approved the final manuscript.

## Pre-publication history

The pre-publication history for this paper can be accessed here:

http://www.biomedcentral.com/1471-244X/10/11/prepub
